# New approach to optimize therapy in type 2 diabetes mellitus: the importance of subclassification

**DOI:** 10.3389/fendo.2025.1710511

**Published:** 2025-11-03

**Authors:** María José Reyes-Medina, Vanesa Cantón-Habas, Francisco Javier Sánchez-Jiménez, Enrique Molina-Hurtado, María del Pilar Carrera-González

**Affiliations:** ^1^ Department of Nursing, Pharmacology and Physiotherapy, Faculty of Medicine and Nursing, University of Córdoba, Córdoba, Spain; ^2^ Maimónides Institute of Biomedical Research of Córdoba (IMIBIC), Córdoba, Spain; ^3^ Reina Sofia University Hospital, Córdoba, Spain; ^4^ Castro del Río Clinic Management Unit, Córdoba Sur Health District, Castro del Río, Córdoba, Spain; ^5^ Experimental and Clinical Physiopathology Research Group CTS−1039, Department of Health Sciences, Faculty of Health Sciences, University of Jaén, Jaén, Spain

**Keywords:** type 2 diabetes mellitus, new subgroups, cluster analysis, treatment algorithm 2024, insulin resistance, beta cell deficiency

## Abstract

Despite advances in diagnosis, monitoring, and pharmacological treatment, type 2 diabetes mellitus continues to be associated with a high number of serious microvascular and macrovascular complications. The objective of this review was to explore the usefulness of subclassification based on patient pathophysiology, its implementation in clinical practice, and its potential to tailor available treatments based on the patient’s pathophysiological profile, in order to evaluate personalized alternatives that optimize the management of this complex disease. In this sense, patients with recently diagnosed type 2 diabetes mellitus could be grouped into seven subgroups: diabetes with pancreatic β-cell deficiency, insulin-resistant diabetes, patients with a combination of deficient insulin secretion and increased resistance, obesity-related diabetes, patients with obesity and a high level of insulin resistance, age-related diabetes, and diabetes with hereditary components. A new algorithm for the stratified diagnostic classification of type 2 diabetes mellitus is presented. According to the reviewed and currently available studies on oral antidiabetics, some drugs may be more effective than others depending on the patient’s subgroup. We propose the administration of insulin or secretagogues in pancreatic β-cell deficiency, thiazolidinediones, SGLT-2 inhibitors or GLP-1 receptor agonists in insulin resistance, and GIP/GLP-1 receptor agonists, GLP-1 receptor agonists or DPP-4 inhibitors depending on the body mass index and the associated risk of hepatic steatosis. Metformin remains recommended as the first-line universal agent across all patient subgroups.

## 1 Introduction

Diabetes Mellitus Type 2 (T2D) is a major chronic disease worldwide, accounting for more than 90% of all diabetes cases (1). This progressive metabolic disorder is characterized by the dysregulation of glycemic homeostasis, leading to chronic hyperglycemia secondary to defects in insulin secretion, insulin resistance, or the coexistence of both processes (2). T2D is a heterogeneous condition with variable clinical manifestations, closely related to factors such as obesity, ethnicity, and family history (3).

Although traditionally considered a disease associated with adulthood and/or old age, it is increasingly diagnosed at an earlier age. This phenomenon is especially concerning since T2D is linked to higher mortality and morbidity compared to the general population (4). Despite advances in diagnosis, monitoring, and pharmacological treatment, this clinical entity continues to be associated with a high number of serious micro and macrovascular complications, such as strokes, acute coronary events, neuropathy, retinopathy, kidney disease, heart failure and its association with cognitive impairment and dementia. In fact, the risk of developing cardiovascular disease (CVD) in patients with T2D is between 2 and 4 times higher, with coronary artery disease (CAD) being the cause of death in 75% and cerebrovascular or peripheral vascular disease in 25% (5).

Thus, in 2021, data recorded in the Institute for Health Metrics and Evaluation confirmed that hyperglycemia causes approximately 11% of deaths from cardiovascular causes (6).

All of this underscores the need for new approaches to early detection, slowing progression and preventing associated complications (7).

Classically, diabetes is divided into several categories, including type 1 diabetes mellitus, type 2 diabetes mellitus and other well-characterized groups such as gestational diabetes, type 3c diabetes, adult autoimmune diabetes (LADA), monogenic and drug-induced diabetes (8). The heterogeneity of type 2 diabetes makes it difficult to identify the specific pathophysiology in each patient, and clinical guidelines do not incorporate subclassification criteria that allow for risk differentiation. Applying a subgroup-based approach could facilitate more precise therapeutic decisions and improve complication prevention.

In this context, studies such as those by Ahlqvist et al. in European populations and Anjana et al. in Asian Indian cohorts have demonstrated, through cluster analysis, that T2D can be subdivided into clinically meaningful groups with distinct pathophysiological profiles and complication risks (9, 10). While these contributions highlight the heterogeneity of the disease, they remain primarily descriptive and do not provide a practical diagnostic algorithm or therapeutic recommendations for each subgroup. The present review expands on this perspective by proposing seven clinically and pathophysiologically based subtypes, accompanied by a stratified algorithm that directly links subclassification with specific pharmacological options, aiming to facilitate the translation of this concept into personalized management in routine clinical practice.

In this sense, establishing empirical subclassifications of T2D would better predict its associated pathology and therefore lead to more specific treatment regimens, improving the prognosis of this disease (11). This goal has been partially achieved in the case of monogenic diabetes, where treatments according to the genetic subtype have shown better results than standard care (12).

In general, therapeutic strategies for T2D are limited, with a regimen adapted to the pathology in which the response to the different classes of antidiabetic agents varies from person to person. Therefore, in addition to evidence-based treatment, it would be interesting to supplement it with a therapeutic strategy based on pathophysiological history, which includes, in turn, non-hypoglycemic drugs to comprehensively address the disease (13, 14). However, to date, there are no prospective studies to support this claim.

The objective of this review is to explore the utility of subclassification in the diagnosis of T2D, its implementation in clinical practice, and its impact on the development of specific pharmacological treatments, in order to assess personalized alternatives that optimize the management of this complex disease.

### 1.1 Deficiency in insulin secretion and insulin resistance

**Figure 1:**
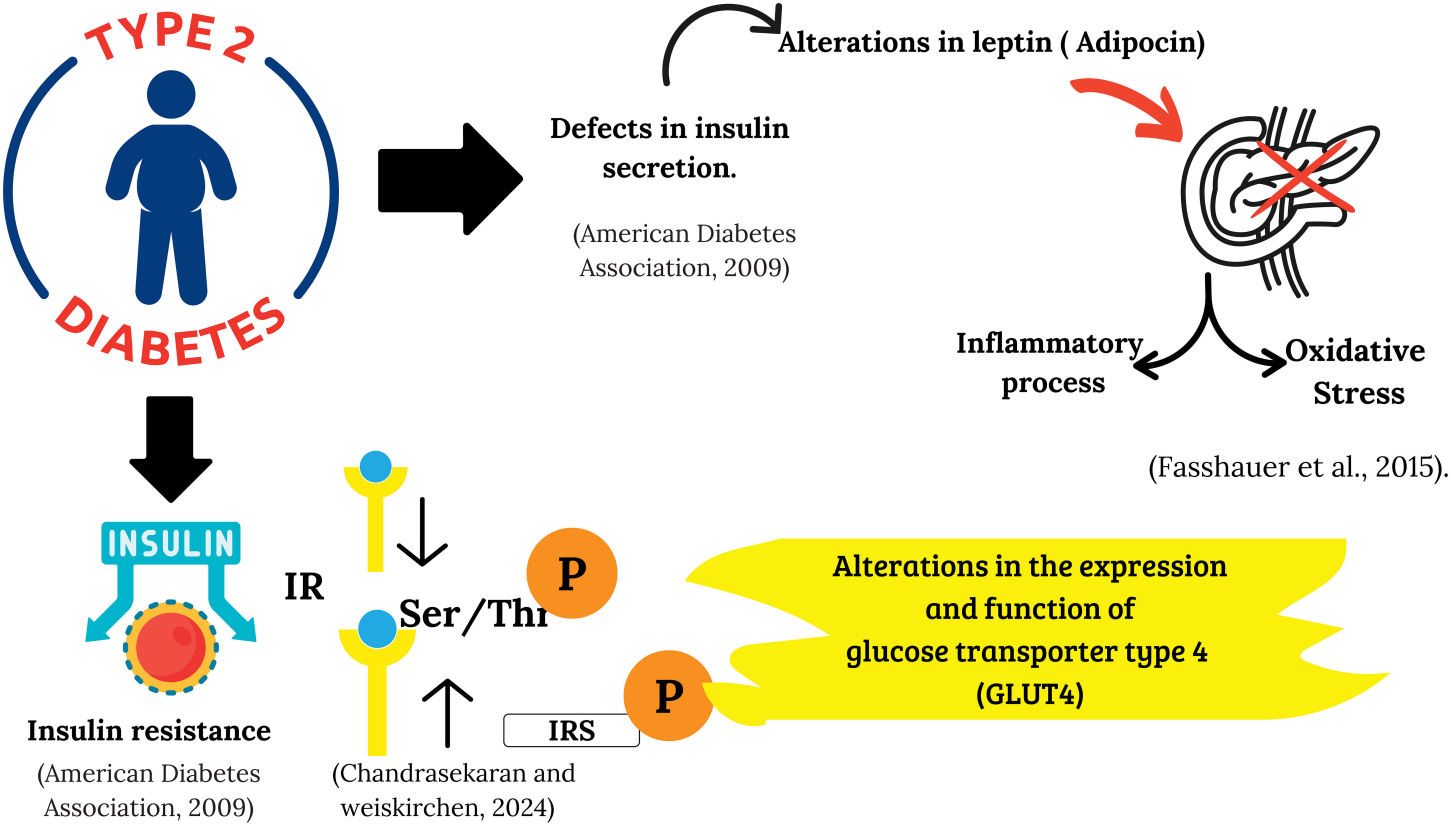
Pathophysiology of type 2 diabetes mellitus: insulin resistance and beta cell dysfunction. The figure provides a detailed description of the pathophysiology of type 2 diabetes: impaired insulin secretion, in a setting of inflammation and oxidative stress, promotes insulin resistance through inhibitory phosphorylations of IRS-1, which impairs insulin signaling. This dysfunction culminates in a reduction in the expression and translocation of the GLUT4 transporter, limiting glucose uptake in muscle and adipose tissue.

There is full consensus in recognizing that the pathophysiology of T2D is related to two processes: the ineffective action of insulin and its deficient secretion, in which multiple signaling pathways from different organs interact (15). Both processes contribute to the development of the disease, resulting in inadequate uptake, storage, and disposal of ingested glucose, along with excessive hepatic glucose production, which culminates in chronic hyperglycemia (16) (**Figure 1**).

### 1.2 Mechanisms associated with pancreatic β-cell dysfunction

Adipose tissue stores fatty acids and maintains energy homeostasis through its communication with organs such as the brain, muscles, liver, and pancreas. When the function of this tissue is modified, there is an alteration in the secretion of cellular signaling molecules, known as adipokines, among which leptin and resistin stand out (17). An increase in leptin levels can induce damage to pancreatic β-cells because it inhibits insulin synthesis, induces inflammatory processes, and generates oxidative stress. On the other hand, resistin, another adipokine secreted by adipose tissue, promotes the release of cytokines, such as interleukin 6 (IL-6) and tumor necrosis factor (TNF), through the activation of nuclear factor kappa B (NF-κB) (18). In contrast, adiponectin, an anti-inflammatory agent that inactivates NF-κB, is elevated in the blood after weight loss in obese individuals, which improves insulin sensitivity (19). The imbalance in the concentration of cytokines and elements that protect the function of pancreatic β-cells can generate functional damage and cell apoptosis (18).

### 1.3 Mechanisms associated with insulin resistance

Insulin resistance (IR) is defined as an alteration in the biological response of target cells to insulin, which translates into a reduction in glucose uptake by muscle and adipose tissues. Among the most common causes of IR are the reduction in the number of insulin receptors (IR) and their catalytic activity, as well as the increase in phosphorylation of serine/threonine (Ser/Thr) residues of the IR and the insulin receptor substrate (IRS). In addition, the decrease in the activity of phosphatidylinositol 3-kinase (PI3K), serine/threonine kinase (Akt), the increase in the activation of tyrosine residue phosphatases (Tyr), and alterations in the expression and function of glucose transporters type 4 (GLUT4) contribute directly to resistance (20).

In patients with obesity, adipose tissue cells release high amounts of IL-6, resistin, and TNF-alpha, cytokines that will alter the phosphorylation of Tyr residues of IRS-1 and the genetic expression of GLUT-4. In addition, these substances activate lipolysis, releasing free fatty acids from the adipocyte, which, when accumulated in the muscle and liver, cause lipotoxicity. In this sense, diacylglycerol (DAG), formed from long-chain acyl-CoA, plays a fundamental role in this process by activating protein kinase C isoforms. This activation alters the phosphorylation of IRS-1 and PI3K, blocking the translocation of GLUT4 and, therefore, reducing glucose uptake (21).

## 2 Heterogeneity of type 2 diabetes

Beyond recognizing T2D as a complex polygenic disease, there is a notable variation in the phenotype of these patients, driven by defects in multiple pathophysiological pathways. Although many of them are not yet fully characterized, it is known that obesity, chronic inflammation, and dysfunctions in lipid metabolism play a crucial role in the pathophysiology, development, and progression of this disease.

### 2.1 Obesity

In Europe and the United States, T2D has been described mainly as a model of processes linked to obesity. As mentioned, ectopic fat accumulation in both skeletal muscle and the liver increases insulin resistance (IR) (22) and impairs insulin action (23). However, the considerable heterogeneity of this pathology represents a challenge for its prevention. This is because, although obesity constitutes an important risk factor, most people with a high body mass index (BMI) do not develop T2D. However, a certain proportion of people with normal weight also develop it (14). In this sense, in relation to ethnic differences, it has been observed that the Indo-Asian population, due to its greater predisposition to the accumulation of visceral fat, has a greater IR despite having a lower BMI. This can probably be related to the lower capacity of subcutaneous fat storage. However, adiposity alone does not fully explain the excessive risk of T2D in these populations (24).

In fact, several studies suggest that this population has a slightly different pathophysiology with an earlier and faster dysfunction of pancreatic β-cells, which positions severe insulin deficiency as the main pathophysiological defect in the development of the disease (10). Because IR and secretory insufficiency are two well-established characteristics of T2D, the working group led by Tanabe et al., suggests that, despite this variability among individuals, the onset and development of diabetes in Asian and non-Asian populations can be divided into two main subtypes: obese insulin-resistant and lean insulin-sensitive (14, 25). This classification reflects the pathophysiological diversity of T2D and suggests the need for personalized approaches in clinical management.

### 2.2 Inflammation

**Figure 2:**
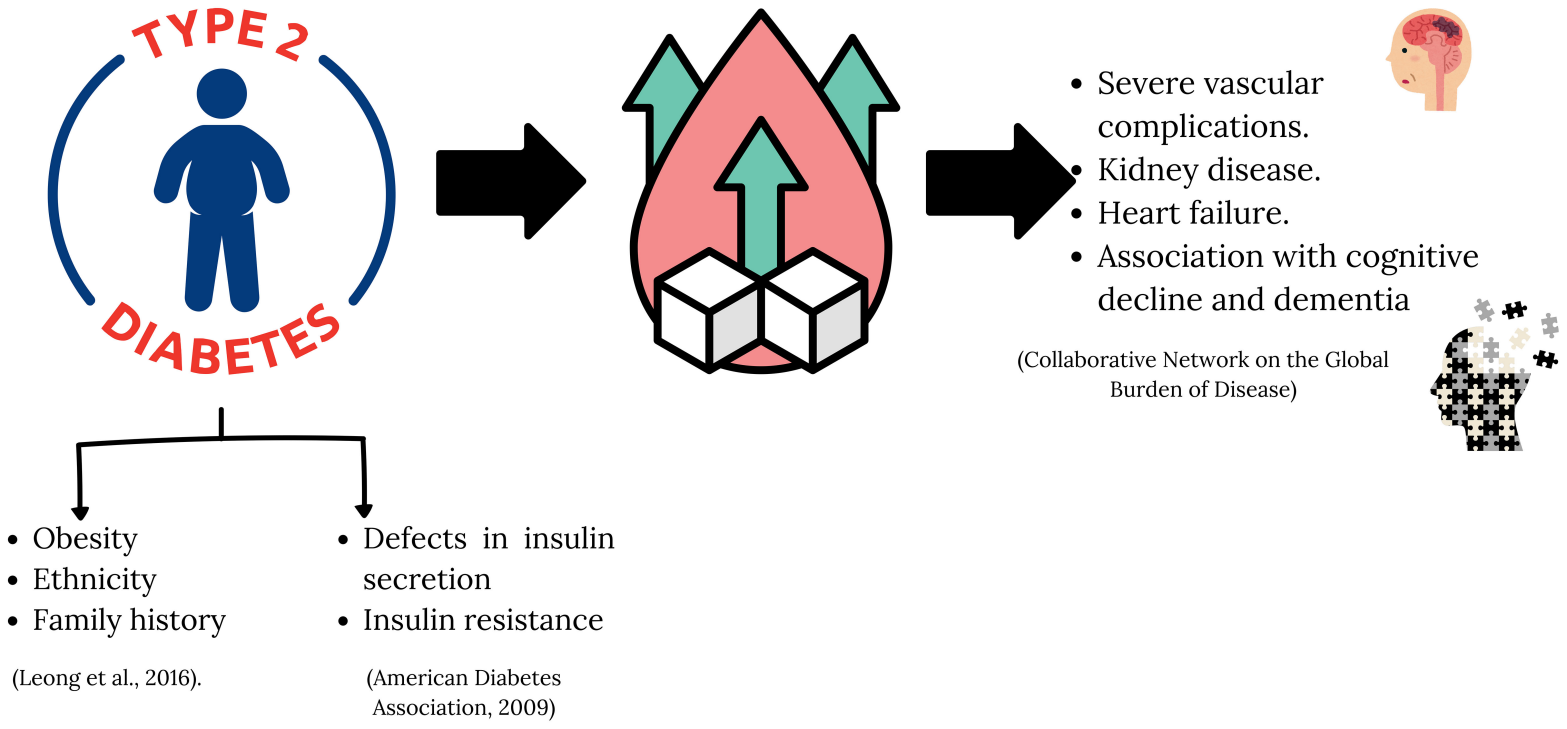
Factors and complications associated with type 2 diabetes mellitus. Type 2 diabetes is influenced by non-modifiable factors such as obesity, ethnicity, and family history, as well as metabolic disorders such as impaired insulin secretion and insulin resistance. Its progression can lead to serious chronic complications, including cardiovascular damage, kidney disease, heart failure, and cognitive decline or dementia.

T2D is a disease associated with subclinical systemic inflammation, which acts as a pathogenic link between the disease and its complications, such as kidney disease, cardiovascular disease, depression, cognitive impairment, and dementia (**Figure 2**).

The immune response and inflammatory modulators are strongly related to pancreatic β-cell dysfunction and IR in patients with T2D. For example, pro-inflammatory cytokines such as interleukin-1β (IL-1β) inhibit insulin secretion and the proliferation of pancreatic β-cells by inducing the transcription and secretion of chemokines (26). Therefore, the inhibition of these inflammatory factors could protect pancreatic β-cells and delay the progression of hyperglycemia (27).

Inflammation and oxidative stress, exacerbated by the imbalance between the production of reactive oxygen species and hyperglycemia, are essential in the progression of diseases such as diabetic nephropathy (DN) (28). Evidence indicates that, despite being a multifactorial disease, the immune response and inflammation are relevant factors. Currently, among the factors related to inflammation, the following stand out: ultra-sensitive C-reactive protein (hs-CRP), toll-like receptor 2 (TLR2), toll-like receptor 4 (TLR4), IAP-1, IL-1β, IL-6, IL-18, TNF-α, NFkB, the Janus kinase/signal transducers and activators of transcription (JAK/STAT) pathway. All of them are linked to signaling pathways of oxidative stress, hyperglycemia and its complications (29).

On the other hand, inflammatory biomarkers, when dysregulated, contribute to states of hypercoagulability and hypofibrinolysis, a fact observed in patients with T2D. The study conducted by Randeria et al., identified elevated levels of anti-inflammatory cytokines (IFN-α, IL-10, Il-4, IL-13, IL-1β, IL-6, IL-8 and sP-selectin, among others) in patients with T2D, a novel finding in the context of this disease (30). These pro-inflammatory signaling pathways and their triggering products appear to be promising biomarkers and therapeutic targets. Currently, anti-inflammatory therapy based on a healthy diet has proven effective by increasing plasma levels of antioxidants, reducing C-reactive protein and mitigating the effect of acute hyperglycemia on inflammation, oxidative stress and endothelial function (31).

### 2.3 Lipid metabolism

Hyperglycemia, dyslipidemia, and hypertension, combined with the duration and type of diabetes, contribute to the distinctive pathophysiology of chronic complications such as diabetic kidney disease, diabetic neuropathy, and retinopathy. Elevated levels of plasma free fatty acids (FFAs) trigger a marked inhibition of insulin mRNA expression, decreasing both glucose-stimulated insulin secretion and insulin content of pancreatic islets (32).

As will be seen later, although there are no drugs specifically approved to treat IR, metformin and thiazolidinediones (TZDs) are sensitizers and increase peripheral glucose utilization (33).

Increased fatty acid metabolism in adipose tissue and lipogenesis in response to elevated glycemia and IR contribute to dyslipidemia in diabetes. Therefore, increased IR leads to overproduction of very low-density lipoprotein cholesterol (VLDL-C) and increased concentration of very low-density lipoproteins (VLDL), which clinically manifests as hypertriglyceridemia. In addition, VLDL-C could stimulate the exchange of triglycerides to cholesterol esters of high-density lipoproteins (HDL) and low-density lipoproteins (LDL), which causes a higher catabolic rate of HDL and the conversion of LDL to small and dense LDL, which are highly atherogenic (34).

These processes also lead to an overproduction of ApoB-containing lipoproteins in the liver, which manifests as hepatic overproduction of VLDL. The atherogenic dyslipidemia associated with these changes is a significant risk factor for the development of cardiovascular disease in patients with T2D. Therefore, it is important to establish interventions to reduce future macrovascular events (35).

**Figure 3:**
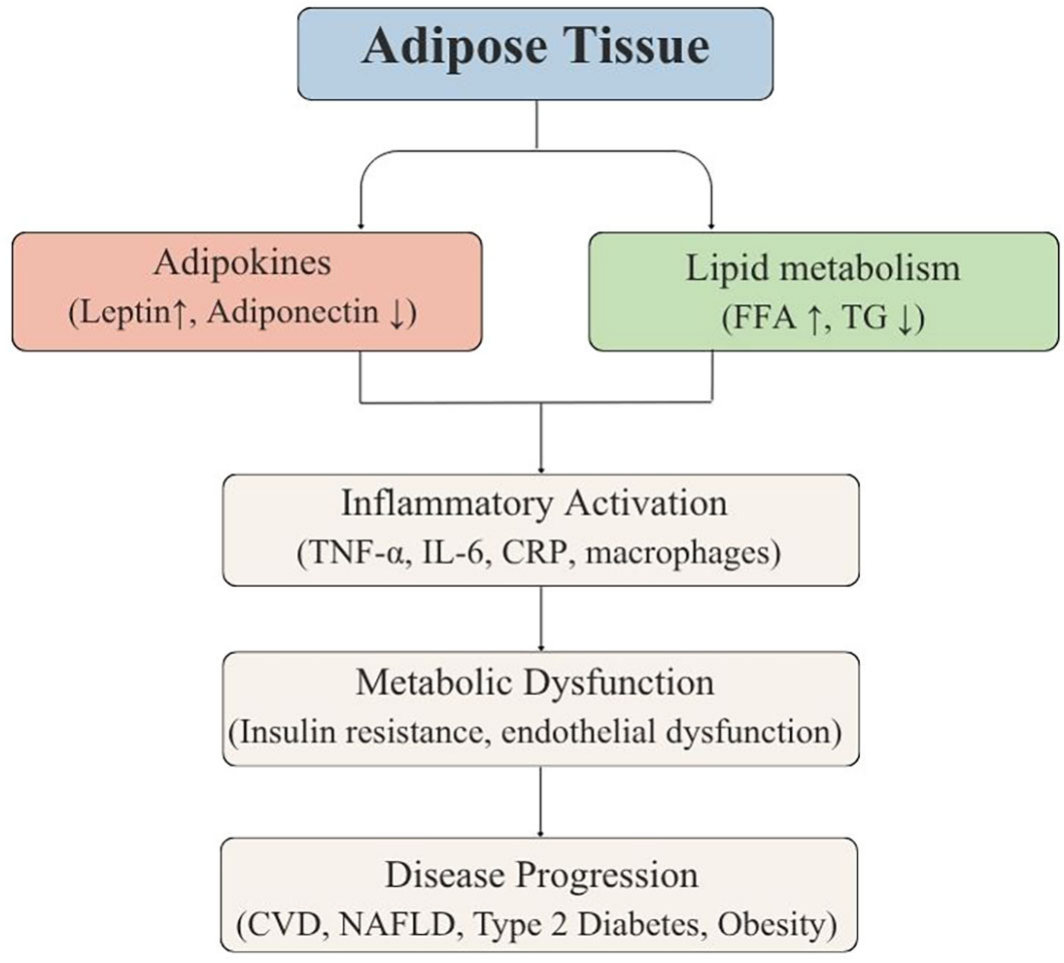
The diagram shows how adipokines modulate inflammation and lipid metabolism, converging on key processes of metabolic dysfunction. FFA: Free atty acids; TG: Triglycerides; TNF-α: Tumor necrosis factor alpha; IL-6: Interleukin 6; CRP: C-reactive protein; CVD: Cardiovascular disease; NAFLD = non-alcoholic fatty liver disease.

These findings reinforce the multifactorial nature of type 2 diabetes, summarized in **Figure 3**, which integrates the role of adipokines, inflammation, and lipid metabolism in metabolic dysfunction.

## 3 Evolution in diagnosis

In clinical practice, diabetes is classified as T2D if the person is over 30 years of age and presents any of the following criteria: overweight or obesity, insidious onset, absence of ketonuria/ketonemia, family history of T2D, history of gestational diabetes. The rest will be considered as other types of diabetes. The absence of a precise classification adapted to the clinic of each patient hinders the development and use of personalized therapy aimed at specific complications of diabetes (36).

The Precision Medicine in Diabetes Initiative (PMDI) was issued in 2018 by the American Diabetes Association (ADA) in coordination with the European Association for the Study of Diabetes (EASD) (ADA/EASD, 2020). The objective of the PMDI is the application of precision medicine in the five fundamental areas of care for this disease (prevention, diagnosis, treatment, monitoring and prognosis) to reduce the growing and enormous prevalence of diabetes worldwide (37).

In this context, numerous studies are focused on stratifying the diagnosis of T2D into homogeneous subgroups based on clinical, genetic and pathophysiological characteristics that may reveal the enormous variability of this pathology (38).

Although current evidence supports the existence of differential subtypes of T2D and their association with variations in clinical outcomes, this information has not yet been integrated into clinical guidelines (39). This may be because research is in preliminary stages and requires further validation before widespread adoption. It is essential to develop and validate subclassification methods that are not only accurate and clinically useful but also cost-effective and applicable in diverse contexts, including those with limited resources. A precision diagnosis could allow treatment to be focused specifically according to the complications associated with the disease, thereby improving patient health and reducing costs for the health sector (40).

### 3.1 Identification of subgroups

Numerous studies confirm the proposal made by Ahlqvist et al., which suggests the possibility of grouping patients into different subgroups and that belonging to a subgroup is more strongly linked to the predisposition of certain complications (9, 11).

The identification of homogeneous groups is carried out through a K-means cluster analysis (CA). Patients with adult-onset diabetes are stratified by applying a data-driven approach, using clinical variables such as the level of glutamate decarboxylase antibodies (GADA), age at diagnosis, BMI, glycated hemoglobin (HbA1c), measures of beta-cell function (HOMA2-B) and insulin resistance (HOMA2-IR). The reviewed studies used homeostasis model assessment indices based on C-peptide (HOMA, or its updated variant, HOMA 2) (41). The analysis, differentiated by sex, carried out by Ahlqvist et al., was performed, for the first time, in a Swedish cohort (n=8980), All New Diabetics in Scania (ANDIS), with prospective data from patient records on the development of complications and the prescription of medication. Five replicable subgroups were identified with significantly different clinical characteristics, disease progression and outcomes. The groups were named severe autoimmune diabetes (SAID), due to the presence of GADA antibodies, severe insulin deficient diabetes (SIDD), severe insulin resistant diabetes (SIRD), mild obesity-related diabetes (MOD) and the fifth group, mild age-related diabetes (MARD) (9).

Due to possible differences between ethnicities, genetic backgrounds and lifestyles, the CA has been replicated in different populations confirming the identification of the non-autoimmune diabetes subtypes of the ANDIS cohort (42). To refine this classification, other clinical parameters that contribute to the progression of diabetes have been included in the analyses, among which are triglycerides (TG), high- and low-density lipoproteins (HDL and LDL), uric acid (UA), waist circumference, estimated glomerular filtration rate (eGFR) and fasting and stimulated C-peptide (10, 28). The result has been the identification of new subgroups. Unlike genetic tests, the use of clinical parameters at the time of diagnosis is an easily applicable method in routine clinical practice to classify diabetes and establish a more individualized treatment plan.

#### 3.1.1 Differential characteristics and proposed clinical algorithm

The subtypes of T2D identified with solidity up to the moment of this review are described below. The risk of damage in different organs and the progression of the disease seem to differ according to the subgroups (11) (**Supplementary Tables 1**, **Tables 1**, **2**).

**Table 1:** Parameter values used to identify subgroups.

Diabetes	Subgrups	TG mmol/L		HDL mmol/L		UA μmol/L
		$\bar{\text{X}}$ (SD)	Median	$\bar{\text{X}}$ (SD)	Median	$\bar{\text{X}}$ (SD)
Pancreatic β cell deficiency	MIDD (43)		1.38 (1,0, 2.01)		1.30 (1.09, 1.52)	
Insulin resistance	UARD (44)	2.1 (1.3)		1.0 (0.3)		436.7 (108)
DUAL: deficiency and insulin resistance	CIRDD (10)	4.68 (0.55)		0.82 (0.21)		
	SIDRD (45)		2.23 (1.48, 3.37)	1.20 (0.29)		
DUAL: obesity and insulin resistance	SOIRD (45)		2.0 (1.54,2.58)	1.22 (0.27)		

TG, Triglycerides; HDL, High density lipoproteins; UA, uric acid.

**Table 2:** Clinical characteristics of the subgroups.

Group	Age (years)	BMI	HbA1c	HOMA2-B/HOMAB	HOMA 2-IR /HOMA IR	TG	HDL	AU
MIDD	‗50	normal weight	controlled	diminished	no resistance	normal	normal	
EOIDD	<50	normal weight	uncontrolled	diminished	no resistance			
LOIDD	‗50	overweight	uncontrolled	diminished	no resistance			
SIDD	‗50	overweight	uncontrolled	diminished	resistant			
EOIRD	<50	obesity	uncontrolled	diminished	signs of resistance			
LOIRD	‗50	overweight	uncontrolled	diminished	signs of resistance			
UARD	‗50	overweight	controlled	good	signs of resistance	elevated	normal	elevated
SIRD	‗50	obesity	controlled	good	resistant			
CIRDD	<50	overweight	uncontrolled	diminished	resistant	elevated	diminished	
SIDRD	‗50	overweight	uncontrolled	diminished	resistant	elevated	normal	
MOD	<50	obesity	uncontrolled	diminished	resistant			
IROD 1	‗50	obesity	uncontrolled	diminished	resistant			
IROD 2	‗50	obesity	controlled	good	resistant			
SOIRD	‗50	overweight	controlled	good	resistant	elevated	normal	
MARD	>65	overweight	controlled	diminished	signs of resistance			
MD								
MDH								
IRD	<50	normal weight	uncontrolled	diminished	No resistance			
SAID								

BMI, Body mass index; HbA1c, Glycosylated hemoglobin; HOMA2-B/HOMAB , Beta cell activity index; HOMA 2-IR/HOMA IR, insulin resistance; TG, Triglycerides; HDL, High density lipoproteins; AU, uric acid.

Reference values: BMI: normal weight (18.5-24.9 kg/m^2^), overweight (25-29.9 kg/m^2^), obesity (‗ 30 kg/m^2^). HbA1c: controlled (¾7%), uncontrolled (>7%). HOMA2-B/HOMAB: diminished (<107), Good (‗ 107). HOMA 2-IR/HOMA IR: No resistance (<1.96), signs of resistance (‗ 1,9 ¾ 3), resistant (>3). TG: normal (<1.7mmol/L), elevated (‗ 1.7 mmol/L). HDL: diminished (<1 mmol/L), normal (1.0-1.55 mmol/L). AU: normal (142–416 mmol/L.), elevated (> 416).

**Figure 4:**
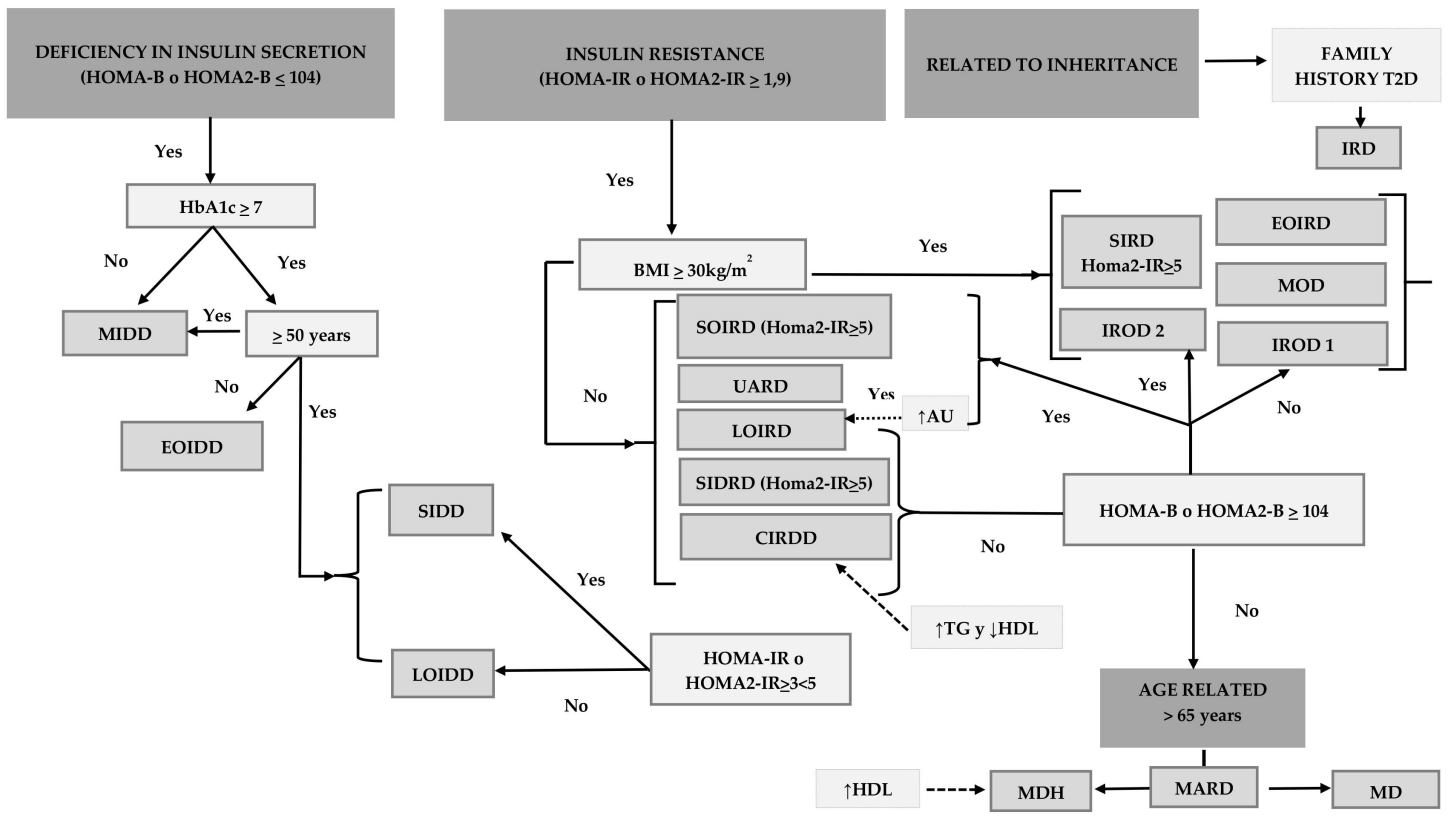
Proposed algorithm for clinical diagnosis of patient with type 2 diabetes mellitus. Figure presents a clinical algorithm that classifies type 2 diabetes subgroups using parameters such as age, HbA1c, body mass index (BMI), HOMA-IR and HOMA-B.

In **Figure 4** we suggest an algorithm for an accurate diagnosis of T2D, based on the underlying pathophysiology of each patient.

##### 3.1.1.1 Diabetes related to deficiency in insulin secretion

###### 3.1.1.1.1 EOIDD AND LOIDD

Early and late-onset insulin-deficient diabetes. Patients included in both groups stand out for having impaired β-cell function and poor metabolic control. They differ in that the LOIDD group had a higher age of onset, greater than 50 years, and a higher prevalence of diabetic kidney disease. In the EOIDD cluster, a high prevalence of diabetic retinopathy stood out (46).

On the other hand, EOIDD and mild insulin-deficient diabetes (MIDD) share similar characteristics, although glycemic control in EOIDD is significantly worse.

###### 3.1.1.1.2 SIDD

Severe diabetes with insulin deficiency. The SIDD group, like the previous subtypes, shows deficient insulin secretion (low HOMA2-B), although with the onset of insulin resistance. It shares similar characteristics with SAID, but without GADA; high values of HbA1c and risk of ketoacidosis (14). Since diagnosis, patients present a high prevalence of diabetic sensorimotor polyneuropathy and cardiac autonomic neuropathy (47). A causal relationship has been observed between abnormal β-cell function and the development of microalbuminuria (MA) (48). Diabetic nephropathy (DN) is preceded by MA, which progresses to proteinuria and reduction of the glomerular filtration rate (GFR) when not treated. Like EOIDD, the SIDD subgroup presents a high incidence of retinopathy (49).

##### 3.1.1.2 Diabetes related to insulin resistance

###### 3.1.1.2.1 EOIRD

Early-onset insulin-resistant diabetes. This group is characterized by mild insulin resistance associated with obesity, poor compensation of pancreatic cells and high prevalence of renal and vascular damage (46).

###### 3.1.1.2.2 LOIRD

Late-onset insulin-resistant diabetes. Compared to EOIRD, patients in LOIRD have a higher age (>60 years), lower BMI, more marked insulin resistance, a somewhat higher HOMA2-β and high prevalence of renal disease and vascular complications. In this group and unlike EOIRD, insulin resistance in addition to having a hepatic origin is also of peripheral origin, influenced by aging, sarcopenia and abnormal fat distribution (46).

###### 3.1.1.2.3 UARD

Diabetes related to uric acid. Xiong et al., used eight variables to perform the groupings, six included in the Ahlqvist study and two new ones, triglyceride (TG) and uric acid (UA) values (9, 44). A new subgroup of T2D was identified, which he called UARD, associated with elevated values of the final product of purine catabolism. There is evidence that UA plays a critical role in diseases such as arterial hypertension (HTA), heart failure (HF) and chronic kidney disease (CKD) (50, 51).

In relation to HOMA values, UARD presents HOMA2-IR values similar to LOIRD, but with correct pancreatic β-cell function, which translates into better metabolic control.

###### 3.1.1.2.4 SIRD

Severe insulin-resistant diabetes. Patients in this group present an age of onset greater than 60 years (similar to LOIRD), a high BMI, moderate to severe insulin resistance with hyperinsulinemia (high HOMA-IR and HOMA2-β), although with better metabolic control, HbA1c lower than that described in the LOIRD, EOIRD groups and similar to UARD (44, 46).

From the moment of diagnosis, patients in SIRD have a higher risk of developing CKD, macroalbuminuria and end-stage renal disease (ESRD), presenting a lower eGFR (40, 49). Insulin resistance, the main characteristic of this group, is an early metabolic alteration in patients with CKD and contributes to the progression of terminal disease and a high cardiovascular risk, due to its multifactorial etiology related to inflammation and obesity (52).

In this sense, the abnormal distribution and function of adiposity, identified in SIRD of the ANDIS cohort, leads to insulin resistance and is associated with a high prevalence of non-alcoholic fatty liver disease (NAFLD), hepatic steatosis and dyslipidemia, with elevated TG values and decreased HDL. In turn, insulin resistance and hyperinsulinemia are significantly associated with the risk of cancer and Alzheimer’s disease (AD), so the risk of developing both diseases also increases in this group (14, 53). In fact, the link between glucose metabolism and AD is increasingly evident, and molecular pathways that characterize this interaction are emerging due to the numerous pathophysiological homologies between both diseases (54).

##### 3.1.1.3 Diabetes with deficiency and insulin resistance

The coexistence of insulin resistance and pancreatic β-cell deficiency, described in the Asian population, seems to be associated with a higher risk of microvascular complications. Below, two subgroups that share intermediate characteristics between the SIDD and SIRD groups are described.

###### 3.1.1.3.1 CIRDD

Combined insulin-resistant and deficient diabetes. The INSPIRED study, led by Anjana et al., identified the coexistence of insulin resistance and deficiency in Asian patients with an early age of onset. CIRDD was characterized by elevated TG levels and reduced HDL cholesterol, which is related to an increased cardiovascular risk. The values of resistance and insulin deficiency were intermediate to those collected in the Scandinavian SIDD and SIRD groups. The presence of dual pathophysiology could be the cause of this more aggressive phenotype, which manifests in the early onset of T2D and predisposes to poor glycemic control. The implementation of therapeutic strategies that combine evaluation tests and agents directed at the underlying causes can help prevent the high prevalence of complications such as retinopathy and diabetic nephropathy observed in CIRDD (10).

###### 3.1.1.3.2 SIDRD

Severe diabetes with deficient and resistant insulin. The SIDRD group is defined by severe insulin deficiency, hypertension, poor metabolic control and insulin resistance, similar to SIRD, despite presenting a lower BMI. Subjects included in this group presented a greater risk of developing CKD, hepatic fibrosis (45) and, therefore, a greater prevalence of cardiovascular events (55). The combination of insulin resistance and deficiency observed in CIRDD and SIDRD may be influenced by the genetic characteristics of the Asian ethnicity, with a lower capacity for insulin secretion and increased insulin resistance compared to Western populations (56).

##### 3.1.1.4 Obesity-related diabetes

###### 3.1.1.4.1 MOD

Mild obesity-related diabetes. The MOD group is characterized by a high BMI, an earlier onset of the disease, and mild insulin resistance, similar to SIDD, although with better pancreatic β-cell function and good metabolic control. The prevalence of complications such as hypertension, NAFLD, and dyslipidemia is lower when compared to the SIDD and SIRD groups (9, 44). Despite this, a public health strategy focused on weight reduction, especially in obese individuals or those with visceral adiposity, could improve insulin sensitivity and resistance, substantially reducing the complications associated with T2D.

##### 3.1.1.5 Obesity-related and insulin-resistant diabetes

###### 3.1.1.5.1 IROD 1

Obese insulin-resistant diabetes 1. The DOLCE study, led by Fedodkina et al., identified a new group of T2D, obese insulin-resistant patients with a disease duration of more than three years. Patients presented similar characteristics to the MOD group, described by Ahlqvist et al., although with worse glycemic control. They presented a high HOMA2-IR index and a reduction in insulin secretion. Its cause could be due to continuous treatment with sulfonylureas (SU), which, although in the short term increases insulin secretion, in the long term decreases it. In addition, IROD 1 is associated with a risk of chronic kidney disease (9, 57).

###### 3.1.1.5.2 IROD 2

Obese insulin-resistant diabetes 2. This long-duration group exhibited high HOMA2-B, good glycemic control, and high insulin resistance, in line with the SIRD group, although with a lower prevalence for all complications than IROD 1. Therefore, two groups of long-duration obese insulin-resistant patients are established: IROD 1 characterized by high HOMA2-IR and reduced HOMA2-B and IROD 2 highlighted by very high HOMA2-B.

The INSPIRED study, which included patients with a diabetes duration of less than 5 years, and the group led by Wang et al., independently identified a group they called IROD, characterized by high BMI, insulin resistance, reduced pancreatic β-cell secretion, and an elevated risk of developing kidney disease, in line with the characteristics of the IROD 1 group (10, 46). Therefore, these findings underscore the need for longitudinal studies to determine whether the difference between the newly diagnosed groups, SIRD and MOD, and the IROD1 and IROD2 groups are due to changes in phenotype over time (57).

###### 3.1.1.5.3 SOIRD

Severe obesity-related diabetes. This group shows a lower BMI than SIRD and MOD, severe insulin resistance, a high level of insulin secretion, good metabolic control, a high risk of developing metabolic-associated fatty liver disease (MAFLD), and a similar risk to SIRD of CKD, although lower than MOD (45, 58).

Among the diagnostic criteria proposed to identify patients with MAFLD are the presence of hepatic steatosis and the coexistence of overweight/obesity or T2D (59).

Therefore, in line with the study conducted by Poustchi et al., we recommend screening to rule out non-alcoholic hepatic steatosis in all patients with T2D, using for its diagnosis the grades calculated by controlled attenuation parameter scores - CAP-3 score and the stages of hepatic fibrosis (F0-F4) by transient elastography (60). It should be noted that the risk of developing NAFLD increases with the degree of hepatic fibrosis and elevated HbA1c levels.

##### 3.1.1.6 Age-related diabetes

###### 3.1.1.6.1 MARD

Mild age-related diabetes. MARD represents a population with favorable BMI and HbA1c values compared to other GADA-negative groups. Patients present a more benign course of the disease, with progressive deterioration of pancreatic β-cells related to aging and associated with a lower risk of developing diabetic complications (9, 61, 62). The IMI-RHAPSODY study identified an additional stratification within the MARD group, establishing two subgroups:

-MDH: has a more beneficial profile. Patients presented high HDL cholesterol values, although eGFR was decreased, probably because these patients are, on average, older.

-MD: patients who do not show remarkable metabolic characteristics (38).

##### 3.1.1.7 Heredity-related diabetes age-related diabetes: IRD

Patients isolated in the CA performed by Xiong et al., had a lower age and BMI, decreased values of HOMA2-B and absence of insulin resistance. In addition, they showed a lower risk of diabetic peripheral neuropathy, hypertension, and chronic kidney disease. A distinctive feature is the high percentage of family history of diabetes, which underscores the crucial role of genetic factors in the pathogenesis of this condition (44).

##### 3.1.1.8 Diabetes related to GADA antibodies

###### 3.1.1.8.1 SAID

Severe autoimmune diabetes. The SAID group, defined by the presence of GADA antibodies, is characterized by a relatively early onset of the disease (<60 years), deficient insulin secretion (HOMA2-B), overweight (>25<30 kg/m2), and poor metabolic control reflected in elevated HbA1c values. Subjects included in this group present a risk of diabetic ketoacidosis, bone fractures, and a higher prevalence and incidence of diabetic retinopathy (14, 49). This group overlaps with both T1D and LADA. Because all patients with antibodies associated with type 1 diabetes, including GADA, should be considered autoimmune (63), this review does not follow the criteria of Ahlqvist et al. (9), and we have not included GADA positivity in the analysis to subclassify type 2 diabetes.

## 4 New perspectives in the treatment of type 2 diabetes

Current recommendations for the management of T2D highlight the importance of personalized treatment based on patient characteristics and associated comorbidities (39, 40). However, the prediction of disease progression or the risk of damage to target organs is not yet fully regulated. In fact, the guidelines clearly indicate the antidiabetic drugs that have been shown to reduce the risk of cardiovascular and renal complications, but other diseases such as NAFLD, retinopathy and neuropathy are relevant conditions that should be considered closely when choosing treatment (63), in order to prevent their progression.

The new classification system, based on clusters, shows the heterogeneity of T2D, based on pathophysiological aspects such as insulin resistance related to obesity or the low secretory capacity of this hormone. The clinical differences between the subgroups suggest that the choice of treatments directed at the underlying metabolic defect could be effective in preventing disease progression and mitigating associated risks.

Based on the treatment evidence that relates T2D to the pathologies mentioned and in trials such as ADOPT and RECORD that evaluated the benefit of different treatments according to the clusters of Ahlqvist, et al., we suggest possible therapeutic strategies for the subgroups described in this review (9). See **Supplementary Table 2**, Suggested therapeutic strategies according to the subgroup of type 2 diabetes.

### 4.1 MIDD

The mild or absent symptoms present among the subjects of this group make this type of diabetes go unnoticed, especially in the Asian population who have an innate deficiency in insulin secretion. Despite the apparent lack of symptomatology, at the time of MIDD diagnosis, the function of pancreatic islets is usually significantly compromised. The finding of this subgroup is of great clinical importance to establish optimal interventions and avoid further deterioration in pancreatic β-cells (43).

Lifestyle modification (healthy diet, regular physical exercise, smoking, stress management, among others) remains the cornerstone of care for people with prediabetes or diabetes at any stage, with the aim of delaying the progression of the disease and future complications. When these measures fail to control glycemia in the initial 3–6 months, it is necessary to prescribe pharmacological therapy. Metformin is currently the most widely recommended first-line pharmacological therapy due to its efficacy, safety profile, and good tolerability. This recommendation is supported by international guidelines (ADA, EASD), which consistently demonstrate reductions in diabetes progression and improvements in glycemic control (37, 64, 65).

In fact, the administration of metformin along with a healthy lifestyle shows additional benefits to prevent disease progression. However, it is important to adjust the dose of metformin according to the patient’s renal function to minimize the risk of lactic acidosis, a rare but serious complication associated with treatment.

### 4.2 EOIDD and LOIDD

The suboptimal glycemic control presented by both groups makes it necessary to intensify treatment compared to the MIDD group.

Considering that the abnormal function of pancreatic β-cells is associated with poor glycemic control, preserving and recovering their function by reducing the excessive workload, seems to be the most effective strategy (66). Initial intensive insulin therapy, defined as the use of this hormone as a first-line hypoglycemic treatment for two or more weeks in patients with newly diagnosed T2D, has been shown to be effective in preserving pancreatic β-cell function. In this sense, the insulinization through multiple daily injections of insulin, for a period of 2 to 3 weeks, not only achieves glycemic control, but also induces glycemic remission, restoring β-cell function (67). Additionally, it improves biomarkers related to inflammation, endothelial function and decreases the risk of ketoacidosis. Furthermore, the observational study by Luo et al., indicates that early insulin therapy may confer cardiovascular benefits, including a reduction in the risk of major events such as stroke and heart failure (68).

Therefore, in view of the characteristics present in EOIDD and LOIDD, this review proposes to initiate treatment with (rapid) insulin for 2 weeks in combination with diet and exercise. If after this time the glycemia values normalize, healthy lifestyle habits should be continued and assess whether treatment with metformin is necessary. Otherwise, and following the guidelines of the American Diabetes Association (ADA) for newly diagnosed patients, with clinical and HbA1c > 10%, we recommend administering basal (slow) insulin along with additional agents or without them (69). Subsequently, as a response is obtained, treatment should be simplified to avoid weight gain and the risk of cardiovascular and microvascular complications (RD) involved in insulin therapy (70).

The EOIDD group showed a high prevalence of RD. This condition is one of the main causes of vision loss developed in response to prolonged hyperglycemia. Therefore, early retinal examinations are recommended to identify lesions of various degrees, such as microaneurysm, hemorrhage and the formation of new vessels, which eventually transform into retinal thickening. A personalized therapy adjusted to the degree of the lesion should be established. Among the treatment modalities are glucocorticoids (dexamethasone, triamcinolone acetonide), intravitreal angiostatic therapy with growth factor inhibitors (bevacizumab), retinal laser therapy and vitrectomy, in the diagnosis of proliferative RD and in diabetic macular edema (42, 71).

### 4.3 SIDD

In this group, insulin treatment would accentuate HOMA2-IR values; therefore, glycemic control with metformin is recommended initially, reserving possible intensification with secretagogues, glinides, or sulfonylureas (SU) for patients who do not achieve an adequate response. If SU therapy is initiated, close monitoring is advised to prevent β-cell function loss. This approach is supported by Saisho et al., who emphasizes the critical importance of preserving functional β-cell mass in the prevention and management of type 2 diabetes. Metformin improves insulin sensitivity and reduces β-cell workload, whereas SU use should be carefully managed to avoid accelerated deterioration of β-cell function (66).

In relation to retinopathy, it is advised to follow the same control and treatment guidelines applied to the EOIDD group, together with incorporation into a physical training program to reduce neuropathic pain. The prescription of exercises in a structured way can delay the incorporation of drugs indicated in ND, among which are tricyclic antidepressants, serotonin and norepinephrine reuptake inhibitors or antiepileptics such as gabapentin and pregabalin (40).

### 4.4 EOIRD

The management of the EOIRD group should focus on reducing BMI to improve insulin sensitivity. Papakonstantinou et al., indicated that lifestyle interventions through moderate weight reduction (7-10%), 150 minutes of moderate intensity exercise per week and approaches to behavioral therapy are effective in treating insulin resistance that causes obesity and preventing the progression of T2D (72). Based on the ADA/EASD consensus guidelines, the indicated line of treatment would be the reduction of body weight and the administration of metformin. This drug, a biguanide, in addition to inhibiting hepatic gluconeogenesis, increases insulin sensitivity and reduces glycemia (73).

In case of not reaching therapeutic objectives, treatment should be intensified with glucagon-like peptide-1 receptor agonists (GLP-1 RA). These drugs (semaglutide, dulaglutide, liraglutide) present a glucose-dependent mechanism of action, stimulating insulin secretion and reducing glucagon secretion in situations of hyperglycemia. In addition, they slow gastric emptying in the early postprandial phase, thus reducing caloric intake and promoting a reduction in body weight and fat mass. In this way, they reduce body weight and body fat mass by reducing caloric intake, which implies an overall reduction in appetite.

GLP-1 receptors are also expressed in organs such as the heart, vascular system, immune system and kidneys. In fact, they exert a beneficial effect on plasma lipids, systolic blood pressure, attenuate the development of atherosclerosis by preventing the progression of the aortic plaque and reducing inflammation in the plaque. The available evidence, according to the meta-analysis conducted by Evans et al., indicates that GLP-1 receptor agonists provide significant cardiovascular and renal benefits compared with DPP-4 inhibitors and basal insulin in patients with type 2 diabetes. Moreover, these agents appear to be a particularly suitable therapeutic option for patients with a BMI > 30 kg/m², as they may confer even greater cardiorenal benefits than basal insulin or DPP-4 inhibitors (74).

### 4.5 LOIRD

Patients included in this group present more impaired renal function than in EOIRD. In this context, the incorporation of sodium-glucose cotransporter inhibitors (SGLT2i), such as empagliflozin, is especially relevant. These drugs increase urinary glucose excretion, reduce HbA1c, body weight, blood pressure and decrease cardiovascular events through an improvement in the content of circulating regenerative cells and the lipid profile (66, 75). These properties make these drugs a useful therapeutic option in the management of patients in LOIRD.

### 4.6 UARD

Therapy aimed at reducing AU levels could be useful for the prevention and treatment of vascular complications in this population (76).

The systematic review carried out by Bletsa et al., provides evidence that allopurinol, a xanthine oxidase inhibitor, can reduce the risk of CVD in patients with T2D by lowering uric acid levels. However, as the authors point out, it is necessary to carry out randomized and placebo-controlled trials to confirm these findings and evaluate their clinical applicability in the patients included in this group (77).

The better glycemic control of this group over EOIRD and LOIRD leads to less intense hypoglycemic treatment, so we suggest allopurinol together with metformin and weight control strategies as therapy to reduce the progression of diabetes.

### 4.7 SIRD

The therapeutic approach for this group focuses on glycemic control guided by the severity of the factors associated with these patients, with special attention to improving insulin sensitivity. Metformin is the first-line drug for SIRD, which is characterized by obesity and severe insulin resistance (14, 37). In addition, intensification of therapy with active interventions is required to reduce hepatic fat, a goal influenced by BMI. According to the Semergen 25 guidelines from the Spanish Society of Primary Care Physicians, pioglitazone is recommended for patients with a BMI< 30 kg/m² (78). In fact, the data-driven subgroup analysis of clinical trial data by Dennis et al., provides robust observational evidence that thiazolidinedione therapy produces greater glycemic benefits in the SIRD group. These drugs enhance insulin sensitivity in skeletal muscle and adipose tissue by increasing the number of GLUT4 transporters in the cell membrane, leading to improved glucose uptake and utilization in tissues, similarly to metformin. However, thiazolidinedione use requires careful cardiovascular monitoring, as it has been associated with an increased risk of heart failure in patients with type 2 diabetes (79).

For patients with a BMI > 30 kg/m2 SGLT2 inhibitors or GLP-1 RA are recommended. SGLT inhibitors are effective in reducing visceral fat (80), ectopic fat (81), improve insulin sensitivity, reduce CKD progression and can reduce obesity-related complications (14). In turn, GLP-1 drugs provide good results in weight reduction, hepatic fat and stroke, so this treatment option is also very beneficial for SIRD (63).

Therefore, according to ADA recommendations, the choice between GLP-1 RA, SGLT2i or combined treatment will depend on the individual characteristics of each patient (associated risk in patients over 55 years of age, atherosclerotic disease, heart failure or kidney disease) (82).

In patients with a high risk of renal and cardiovascular deterioration, the treatment approach is more complex, with the need to combine the aforementioned antidiabetics with non-hypoglycemic drugs, if the patient has not had them indicated. The reduction of albuminuria with inhibitors of the renin-angiotensin-aldosterone system (RAASi) is key to improving renal outcomes and constitutes, together with blood pressure control, the cornerstone of diabetic nephropathy treatment (83). However, sometimes, it is necessary to further intensify treatment. In this sense, sulodexide, a purified mixture of sulfated glycosaminoglycans of low molecular weight heparin, provides a renoprotective benefit through its ability to reduce urinary albumin excretion rates, so it occupies an important place in the treatment for the prevention of end-stage renal disease (84).

### 4.8 CIRDD

The more aggressive phenotype of this group characterized by difficult-to-control hyperglycemia, a product of the combination of insulin deficiency and resistance, together with overweight, dyslipidemia and markedly significant risks of cardiovascular, renal and retinopathy disease, makes it necessary to intensify treatment compared to SIDD and SIRD. Among the therapies considered for patients with high cardiovascular risk, metformin together with SGLT-2i and/or GLP1 RA stands out. In case glycemic control is not sufficient, treatment should be intensified with the incorporation of another oral drug or with basal insulin (78).

Regarding lipid control, SGLT2i improve diabetic dyslipidemia by reducing triglycerides and increasing HDL cholesterol (85). The American Diabetes Association (ADA) indications for the treatment of blood cholesterol in diabetes suggest considering the administration of moderate doses of statins to patients with diabetes mellitus between 40 and 75 years, even without associated cardiovascular risk factors. Therefore, if therapeutic and lifestyle measures do not work, it should be considered to prescribe moderate doses of statins 10–20 mg of atorvastatin, 5–10 mg of rosuvastatin, 20–40 mg of simvastatin or 40–80 mg of pravastatin (64).

It is recommended to follow the same guidelines for control and treatment of retinopathy as the EOIDD and SIDD groups, ensuring regular evaluation and implementation of timely interventions when necessary.

### 4.9 SIDRD

In this group, metformin continues to be the treatment of choice together with SGLT2i to reduce cardiorenal risk and glycemia. According to ADA recommendations, insulin therapy should be initiated if HbA1c is ≥ 10% or glycemia ≥ 300 mg/dL. In this context, the early use of basal insulin degludec or insulin glargine-300 is proposed, prioritizing them over glargine-100 due to their ability to reduce the risk of hypoglycemia, both general and nocturnal (86). In turn, it is recommended to prescribe RAASi treatment in cases of elevated blood pressure to prevent the risk of deterioration at the associated renal level. The possibility of incorporating sulodexide and statins should also be evaluated.

In relation to the risk of hepatic fibrosis, in addition to lifestyle modifications, such as weight loss, adherence to the Mediterranean diet and regular physical activity, current international guidelines suggest pharmacological treatment with vitamin E and pioglitazone for NAFLD (87).

### 4.10 MOD

Weight loss along with lifestyle changes can significantly improve glucose homeostasis and cardiometabolic risk factors in patients with T2D, although these strategies are not effective in the long term (88). In patients with high BMI, novel antidiabetic agents, particularly tirzepatide, a dual GIP/GLP-1 receptor agonist, as well as GLP-1 receptor agonists (semaglutide, dulaglutide, liraglutide), represent the most effective pharmacological strategy for managing type 2 diabetes and obesity insufficiently controlled by lifestyle interventions, as recommended by the Semergen 25 guidelines and supported by evidence from meta-analysis of randomized controlled trials conducted by Karagiannis et al. (78, 89). These agents not only promote significant weight loss but also improve insulin resistance, blood pressure, and plasma lipid profiles, including LDL, HDL, and triglycerides (90).

In case of needing an intensification in treatment, dipeptidyl peptidase 4 inhibitors (DPP4i) are recommended, which act by inhibiting the enzyme that blocks glucagon-like peptide type 1 (GLP1), which allows it to accumulate and remain longer in the body. This, in turn, stimulates insulin secretion after ingestion (the so-called incretinic effect), while at the same time reducing the counter-regulatory hormone, glucagon, and thus decreases glucose production by the liver. DPP4i are characterized by a neutral effect on weight, in the risk of hypoglycemia and in cardiovascular risk, so their indications are postulated to be wider than SU (91, 92).

### 4.11 IROD 1 and 2

The characteristics shared with MOD suggest that patients in the IROD 1 group would benefit from the same established treatment mentioned above. Regarding the IROD 2 group, the study conducted by Fedotkina et al., indicates that treatment may be more flexible, with a smaller number of patients in treatment with insulin, SU and a higher percentage of patients without pharmacological treatment, compared to IROD 1 (57). However, due to the high BMI (> 30 kg/m2) we advise, following the recommendations of Semergen 25, therapy with metformin, SGLT2i/GLP RA or the combination of both (78).

### 4.12 SOIRD

The features of this group lead to a therapy focused on decreasing BMI and improving insulin sensitivity. Therefore, the indicated drugs would be the combination of metformin and pioglitazone. In case of not achieving the proposed objectives and/or presence of metabolic liver disease, the recommended treatment is GLP1 RA/tirzepatide together with SGLT2i (37, 78).

### 4.13 MARD (MDH and MD)

In the case of patients with advanced age and frailty, predominant characteristics of this group, the treatment of choice continues to be metformin. However, it should be taken into account that this drug can intensify side effects over time. In addition, the deterioration of renal function, common in these patients, could lead to toxic levels of metformin. Also, congestive heart failure and liver failure are relative contraindications for treatment with this biguanide due to the increased risk of lactic acidosis (93).

The ADOPT and RECORD trials, on the benefits of differential treatment in subgroups, found a better response when MARD received a sulfonylurea. Despite these results and due to the associated risk of hypoglycemia, these secretagogues are discouraged in frail patients (94).

DPP-4 inhibitors belong to the group of incretin derivatives and have specific advantages for use in association and in special situations. Their low risk of hypoglycemia and weight gain together with the antioxidant and anti-inflammatory effects, which attenuate the cardiovascular complications of diabetes, constitute an effective therapeutic option in frail patients (92, 95). In the event of needing intensification of treatment, the indicated drugs are SGLT2 inhibitors (78).

### 4.14 IRD

In this group, as in others, metformin is the drug of choice. However, the atypical characteristics cited in the description of the group induce research into prospective strategies, such as genome association studies, to identify patients more accurately and quickly. This would allow for a more personalized therapy tailored to individual needs (37).

### 4.15 SAID

Insulin treatment is essential to prevent life-threatening complications in GADA+ patients. In most cases, multiple daily injections are required to achieve and maintain strict glycemic control, reflecting the progressive decline in β-cell function characteristic of this condition (96).

**Supplementary Table 3**, provides a comprehensive summary of the main diabetes subgroups analyzed in this review, highlighting their clinical characteristics, associated risks for disease progression, and recommended therapeutic strategies. This synthesis offers a concise reference tool to facilitate comparison and evaluation across the different subtypes.

## 5 Conclusions

The use of reliable and easily measurable clinical parameters facilitates the diagnosis of T2D by grouping patients with similar pathophysiological characteristics. This stratification evidences the heterogeneity of this population in aspects such as obesity, inflammation and lipid metabolism, allowing to predict the risk of associated complications and optimize therapeutic strategies from early stages of the disease. Therefore, subclassification can be an appropriate tool to improve control and prevent the progression of T2D.

Newly diagnosed patients can be grouped into seven subgroups: diabetes with deficiency of pancreatic β-cells, insulin-resistant diabetes, patients who combine deficient insulin secretion with an increase in resistance, obesity-related diabetes, patients with obesity and a high rate of insulin resistance, age-related and heredity-related diabetes.

Based on the results achieved in the reviewed and currently available studies on antidiabetics, some drugs may be more appropriate for certain subgroups of patients. Metformin continues to be the recommended first-line agent for all the analyzed clusters. Patients with insulin deficiency may benefit from treatment with injections of this hormone or with secretagogues depending on HOMA2-IR and HbA1c values. In insulin resistance, a thiazolidinedione, SGLT-2i or GLP-1 RA is recommended depending on BMI and the risk of CKD. In obese patients with T2D, GIP/GLP-1 RA, GLP-1 RA or DPP-4i are preferred depending on BMI and the associated risk of hepatic fat.

In turn, through this review we highlight the importance of continuous diagnosis and monitoring through functional tests (blood, urine and image analysis) to assess the cardiovascular, neurological, renal and retinal status to adapt treatment according to the patient’s clinical characteristics and their comorbidities.

In conclusion, the subclassification of T2D offers a promising perspective to achieve a more personalized therapy with better health outcomes. Future multi-ethnic longitudinal studies and interventional studies are needed to validate subclassification and integrate it into practice.
