# Supplementary material for: New approach to optimize therapy in type 2 diabetes mellitus: the importance of subclassification
Source: Front Endocrinol (Lausanne). 2025 Nov 3;16:1710511. doi: 10.3389/fendo.2025.1710511 (PMC12620229; doi:10.3389/fendo.2025.1710511)
Supplement: Supplementary file 1 [file Table1.docx]

**Supplementary Table 1.** Parameter values ​​used to identify subgroups

| **DIABETES** | **SUBGRUPS** | **AGE**  **X̄ (SD)**  **Years** | **BMI**  **X̄ (SD)**  **kg/m^2^** | **HbA1c**  **X̄ (SD)**  **%** | **HOMA2-B** | | **HOMA2-IR** | | **HOMA-B**  **Median** | **HOMA-IR**  **Median** |
| --- | --- | --- | --- | --- | --- | --- | --- | --- | --- | --- |
|  |  |  |  |  | **X̄ (SD)** | **Median** | **X̄ (SD)** | **Median** |  |  |
| **Pancreatic β cell deficiency** | MIDD ^(63)^ | 57.13 (7.41) | 22.87 (2.58) | 6.23 (0.58) |  |  |  |  | 41.86  (28.65, 57.56) | 1.62  (1.19, 2.16) |
|  | EOIDD ^(43)^ | 47.4 (12.2) | 24.8 (2.5) | 10.3  (1.9) |  | 40.1  (27.5, 61.4) |  | 1.2  (0.8, 1.7) |  |  |
|  | LOIDD ^(43)^ | 53.1 (7.2) | 25.7 (2.6) | 10.0  (1.7) |  | 46.9  (31.9, 65.5) |  | 1.4  (1.0, 2.0) |  |  |
|  | SIDD ^(9)^ | 56.74 (11.14) | 28.86 (4.77) | 11.50 (3.9) | 47.64 (28.93) |  | 3.18 (1.73) |  |  |  |
| **Insulin resistance** | EOIRD ^(43)^ | 36.7 (9.4) | 33.1 (3.3) | 10.1  (1.7) |  | 58.1  (37.8, 85.6) |  | 1.9  (1.4, 2.6) |  |  |
|  | LOIRD ^(43)^ | 63.8  (9.3) | 27.3  (3.3) | 9.3  (1.8) |  | 74.2  (45.1, 109.9) |  | 2.3  (1.9, 3.0) |  |  |
|  | UARD ^(47)^ | 51.4  (10.8) | 25.9  (4.3) | 7  (1.4) | 129  (66.6) |  | 2.1  (1.2) |  |  |  |
|  | SIRD ^(9)^ | 65.25 (5.24) | 33.85 (5.24) | 7  (3.96) | 150.47 (47.20) |  | 5.54 (2.74) |  |  |  |
| **DUAL: deficiency and insulin resistance** | CIRDD ^(10)^ | 42.1  (9.8) | 26.5  (3.1) | 9.1  (1.9) | 100.8  (51.5) |  | 4.1  (1.5) |  | 64.5  (37.5) | 3.8  (1.9) |
|  | SIDRD ^(53)^ | 59.9  (10.2) | 26.4  (3.6) | 10.6  (1.6) |  | 19.3  (11.3, 29.8) |  | 5.2  (3.1, 7.8) |  |  |
| **Related to obesity** | MOD ^(9)^ | 48.96 (9.54) | 35.71 (5.43) | 7.4  (3.60) | 95.3 (32.45) |  | 3.35 (1.21) |  |  |  |
| **DUAL: obesity and insulin resistance** | IROD 1^(56)^ | 62.1  (7.9) | 36.4  (4.4) | 9.6  (1.7) | 65.7  (27.8) |  | 3.5  (1.1) |  |  |  |
|  | IROD 2 ^(56)^ | 61.7  (8.4) | 32  (4.5) | 7  (1.1) | 139.4  (38.8) |  | 3.1  (1.1) |  |  |  |
|  | SOIRD ^(53)^ | 62.9  (9.0) | 29.8  (3.5) | 6.4  (0.7) |  | 148.9  (116.8, 202.7) |  | 5.6  (4.2, 7.1) |  |  |
| **Age related** | MARD ^(9)^ | 67.37 (8.55) | 27.94 (3.44) | 6.7 (3.10) | 86.59 (26.37) |  | 2.55 (0.84) |  |  |  |
|  | MD ^(38)^ |  |  |  |  |  |  |  |  |  |
|  | MDH ^(38)^ |  |  |  |  |  |  |  |  |  |
| **Related to inheritance** | IRD ^(47)^ | 42,6 (6,1) | 23,0  (2,5) | 8,0  (1,4) | 44,8  (25,3) |  | 1,1  (0,6) |  |  |  |
| BMI= Body mass index, HbA1c= Glycosylated hemoglobin, HOMA2-B / HOMAB = Beta cell activity index, HOMA 2-IR/HOMA IR= insulin resistance, TG= Triglycerides, HDL= High density lipoproteins | | | | | | | | | | |
