# Supplementary material for: New approach to optimize therapy in type 2 diabetes mellitus: the importance of subclassification
Source: Front Endocrinol (Lausanne). 2025 Nov 3;16:1710511. doi: 10.3389/fendo.2025.1710511 (PMC12620229; doi:10.3389/fendo.2025.1710511)
Supplement: Supplementary file 2 [file Table2.docx]

**Supplementary Table 2.** Suggested therapeutic strategies according to the subgroup of type 2 diabetes mellitus

| **GROUP** | **FIRST CHOICE DRUGS** | **THERAPEUTIC ALTERNATIVE OR FIRST INTENSIFICATION** | **RECOMMENDATIONS** |
| --- | --- | --- | --- |
| **MIDD** | Healthy living habits | Metformin  2nd alternative: SU/glinidine | Close monitoring as in prediabetes to avoid progress |
| **EOIDD** | Rapid insulin (2 weeks)  Metformin | Slow insulin and Metformin | Control of retina and kidney function |
| **LOIDD** | Rapid insulin (2 weeks)  Metformin | Slow insulin and Metformin | Control of retina and kidney function |
| **SIDD** | Metformin | Secretagogues (SU/glinidines) | Retinal control and physical training |
| **EOIRD** | Intensive exercise, behavioral therapy and metformin | arGLP-1 | Renal and vascular control |
| **LOIRD** | Metformin,  iSGLT2 |  | Renal and vascular control |
| **UARD** | Metformin  Allopurinol |  | Vascular control |
| **SIRD** | Metformin | If BMI < 30 kg/m2 = pioglitazone  If BMI > 30 kg/m2 = iSGLT2/arGLP-1 or both | Renal and cardiovascular control and monitor NAFLD risk.  RAASi and Sulodexine |
| **CIRDD** | Metformin  iSGLT2 + arGLP-1 | Basal insulin | Control of retina, kidney function, cardiovascular and dyslipidemia.  Consider treatment with statins |
| **SIDRD** | Metformin  iSGLT2 | Basal insulin | Cardiorenal control and liver fibrosis  RAASi  Sulodexine  Vitamina E + pioglitazone in NAFLD |
| **MOD** | Metformin  Tirzepatide or arGLP-1 | iDPP-4 | Control hypertension, NAFLD and dyslipidemia  Withdrawal SU if you have them |
| **IROD 1** | Metformin  Tirzepatide or arGLP-1 | iDPP-4 | Control hypertension, NAFLD and dyslipidemia |
| **IROD 2** | Metformina  iSGLT2/ arGLP-1 | Considerar triple terapia | Kidney and cardiovascular risk control |
| **SOIRD** | Metformin  Pioglitazone | Withdrawal pioglitazone and prescribe iSGLT2 and ARGLP-1 or tirzepatide | Kidney risk control and NAFLD/MAFLD |
| **MARD** | Metformin + iDPP4 | iSGLT2 | Close monitoring for impaired kidney function |
| **IRD** | Metformin | Depending on the characteristics |  |
| **All treatments include HEALTHY LIVING HABITS.**  SU: Sulfonylurea, ar GLP-1: glucagon-like peptide 1 receptor agonists, iSGLT-2: Sodium-glucose cotransporter type 2 (iSGLT2) inhibitors, BMI: Body mass index, NAFLD: non-alcoholic fatty liver disease, RAASi: inhibitors of the renin-angiotensin-aldosterone system, DPP4i: dipeptidyl peptidase 4 inhibitors, MAFLD: metabolic-associated fatty liver disease. | | | |
