# Supplementary material for: New approach to optimize therapy in type 2 diabetes mellitus: the importance of subclassification
Source: Front Endocrinol (Lausanne). 2025 Nov 3;16:1710511. doi: 10.3389/fendo.2025.1710511 (PMC12620229; doi:10.3389/fendo.2025.1710511)
Supplement: Supplementary file 3 [file Table3.docx]

**Supplementary Table 3.** Summary of Diabetes Subgroups: Characteristics, Risks, and Therapy Guidelines

| **GROUP** | **CLINICAL FEATURES** | **DEVELOPMENT RISK** | **FIRST CHOICE DRUGS** |
| --- | --- | --- | --- |
| **MIDD** | Middle-aged (> 50 years)  Normal weight  Insulin secretion adequate  Reduced β-cell function  Not insulin resistant | High predisposition to pancreatic β-cell deficiency | Healthy living habits |
| **EOIDD** | Younger adults (<50 years)  Normal weight  HbA1c uncontrolled  Reduced β-cell function  Not insulin resistant | High prevalence of diabetic retinopathy | Rapid insulin (2 weeks)  Metformin |
| **LOIDD** | Middle-aged (> 50 years)  Overweight  HbA1c uncontrolled  Reduced β-cell function  Not insulin resistant | Risk of Diabetic kidney disease | Rapid insulin (2 weeks)  Metformin |
| **SIDD** | Middle-aged (> 50 years)  Overweight  HbA1c uncontrolled  Reduced β-cell function  Insulin resistant | Risk of microvascular and neurological complications | Metformin |
| **EOIRD** | Younger adults (<50 years)  Obesity  HbA1c uncontrolled  Reduced β-cell function  Sign of insulin resistance | High prevalence of renal and vascular damage | Intensive exercise, behavioral therapy and metformin |
| **LOIRD** | Middle-aged (> 50 years)  Overweight  HbA1c uncontrolled  Reduced β-cell function  Sign of insulin resistance | High prevalence of renal and vascular damage | Metformin,  iSGLT2 |
| **UARD** | Middle-aged (> 50 years)  Overweight  HbA1c controlled  Insulin secretion adequate  Sign of insulin resistance  High triglycerides and uric acid | Hidney damage, cardiovascular and metabolic disease. | Metformin  Allopurinol |
| **SIRD** | Middle-aged (> 50 years)  Obesity  HbA1c controlled  Insulin secretion adequate  Insulin resistance | High risk of renal, cardiovascular and metabolic complications, with dyslipidemia and increased risk of cancer and Alzheimer's disease | Metformin |
| **CIRDD** | Younger adults (<50 years)  Overweight  HbA1c uncontrolled  Reduced β-cell function  Insulin resistance  Elevated triglycerides and decreased HDL | Cardiovascular, renal and microvascular complications, | Metformin  iSGLT2 + arGLP-1 |
| **SIDRD** | Middle-aged (> 50 years)  Overweight  HbA1c uncontrolled  Reduced β-cell function  Insulin resistance  High triglycerides | Kidney, liver and cardiovascular complications | Metformin  iSGLT2 |
| **MOD** | Younger adults (<50 years)  Obesity  HbA1c uncontrolled  Reduced β-cell function  Insulin resistance | Moderate risk of metabolic, cardiovascular, and liver complications, generally lower than in SIDD and SIRD, especially if good glycemic control and weight reduction are maintained. | Metformin  Tirzepatide or arGLP-1 |
| **IROD 1** | Middle-aged (> 50 years)  Obesity  HbA1c uncontrolled  Reduced β-cell function  Insulin resistance | Renal and metabolic complications | Metformin  Tirzepatide or arGLP-1 |
| **IROD 2** | Middle-aged (> 50 years)  Obesity  HbA1c controlled  Insulin secretion adequate  Insulin resistance | Kidney and cardiovascular risk | Metformina  iSGLT2/ arGLP-1 |
| **SOIRD** | Middle-aged (> 50 years)  Overweight  HbA1c controlled  Insulin secretion adequate  Insulin resistance  High triglycerides | Kidney and liver risk | Metformin  Pioglitazone |
| **MARD** | Aged over 65 years  Overweight  HbA1c controlled  Reduced β-cell function  Sign of insulin resistance | Metabolic, renal and cardiovascular complications | Metformin + DPP4i |
| **IRD** | Younger adults (<50 years)  Normal weight  HbA1c uncontrolled  Reduced β-cell function  Not insulin resistant | Low risk of metabolic, renal and cardiovascular complications, with greater influence of genetic factors in the pathogenesis of diabetes. | Metformin |
| **All treatments include HEALTHY LIVING HABITS.**  HbA1c= Glycosylated hemoglobin; iSGLT-2: Sodium-glucose cotransporter type 2 (iSGLT2) inhibitors; ar GLP-1: glucagon-like peptide 1 receptor agonists; DPP4i: dipeptidyl peptidase 4 inhibitors | | | |
